# Biological Distribution of Orally Administered [^123^I]MIBG for Estimating Gastrointestinal Tract Absorption

**DOI:** 10.3390/pharmaceutics14010061

**Published:** 2021-12-28

**Authors:** Masato Kobayashi, Asuka Mizutani, Yuka Muranaka, Kodai Nishi, Hisakazu Komori, Ryuichi Nishii, Naoto Shikano, Takeo Nakanishi, Ikumi Tamai, Keiichi Kawai

**Affiliations:** 1 Faculty of Health Sciences, Institute of Medical, Pharmaceutical and Health Sciences, Kanazawa University, 5-11-80 Kodatsuno, Kanazawa 920-0942, Japan; mizutani.a@staff.kanazawa-u.ac.jp (A.M.); yukarisa93@stu.kanazawa-u.ac.jp (Y.M.); kei@mhs.mp.kanazawa-u.ac.jp (K.K.); 2 Department of Radioisotope Medicine, Atomic Bomb Disease Institute, Nagasaki University, 1-12-4 Sakamoto, Nagasaki 852-8523, Japan; koudai@nagasaki-u.ac.jp; 3 Faculty of Pharmaceutical Sciences, Institute of Medical, Pharmaceutical and Health Sciences, Kanazawa University, Kakuma, Kanazawa 920-1192, Japan; hkomori@p.kanazawa-u.ac.jp (H.K.); tamai@p.kanazawa-u.ac.jp (I.T.); 4 Department of Molecular Imaging and Theranostics, Institute for Quantum Medical Science Quantum Life and Medical Science Directorate, National Institutes for Quantum Science and Technology (QST), 4-9-1 Anagawa, Inage, Chiba 263-8555, Japan; nishii.ryuichi@qst.go.jp; 5 Department of Radiological Sciences, Ibaraki Prefectural University of Health Sciences, 4669-2 Ami, Inashiki, Ibaraki 300-0394, Japan; sikano@ipu.ac.jp; 6 Faculty of Pharmacy, Takasaki University of Health and Welfare, 60 Nakaorui-machi, Takasaki 370-0033, Japan; nakanishi@takasaki-u.ac.jp; 7 Biomedical Imaging Research Center, University of Fukui, 23-3 Matsuokashimoaizuki, Eiheiji, Fukui 910-1193, Japan

**Keywords:** [^123^I]MIBG, SPECT, imaging, drug transporters, gastrointestinal tract absorption

## Abstract

Gastrointestinal tract absorption of cationic anticancer drugs and medicines was estimated using whole-body imaging following oral [^123^I]MIBG administration. [^123^I]MIBG was added to cultures of human embryonic kidney (HEK)293 cells expressing human organic anion transporting polypeptide (OATP)2B1, carnitine/organic cation transporter (OCTN)1 and OCTN2, and organic cation transporter (OCT)1, OCT2, and OCT3 with and without cimetidine (an OCTN and OCT inhibitor) and L-carnitine (an OCTN inhibitor). Biodistribution analyses and single-photon emission computed tomography (SPECT) imaging in normal and dextran sodium sulfate (DSS)-induced experimental colitis mice were conducted using [^123^I]MIBG with and without cimetidine. [^123^I]MIBG uptake was significantly higher in HEK293/OCTN1, 2, and OCT1-3 cells than in mock cells. Uptake via OCTN was inhibited by L-carnitine, whereas OCT-mediated uptake was inhibited by cimetidine. Biodistribution analyses and SPECT imaging studies showed significantly lower accumulation of [^123^I]MIBG in the blood, heart, liver, and bladder in DSS-induced experimental colitis mice and mice with cimetidine loading compared with normal mice, whereas significantly higher accumulation in the stomach and kidney was observed after [^123^I]MIBG injection. [^123^I]MIBG imaging after oral administration can be used to estimate gastrointestinal absorption in the small intestine via OCTN and/or OCT by measuring radioactivity in the heart, liver, and bladder.

## 1. Introduction

Iodine-123 labeled *m*-iodobenzylguanidine ([^123^I]MIBG), an analog of the adrenergic neurotransmitter norepinephrine, has been used as a cardiac [1] and adrenomedullary scintigraphic agent [2] since the early 1980 s. [^123^I]MIBG is primarily transported into cardiac and neuroblastoma cells via organic cation transporter (OCT)1-3, including the norepinephrine transporter [3,4].

Many anticancer drugs (e.g., imatinib) and medicines (e.g., metformin, cimetidine, procainamide) are incorporated into the blood from the small intestinal epithelial cell membrane via solute carrier (SLC) transporters. At the small intestinal epithelial cell membrane in humans, organic anion transporting polypeptide (OATP)2B1 (*SLCO2B1*), organic cation/carnitine transporter (OCTN)1 (*SLC22A4*), OCTN2 (*SLC22A5*), organic cation transporter (OCT)1-3, and peptide transporter 1 (*SLC*15A1) isoforms are expressed primarily as SLC transporters [5,6,7,8,9]. Although the enterocyte membrane localization of the transporters, especially OATP2B1 and OCT1, changes along the gastrointestinal tract [10], the SLC transporters OATP2B1, OCTN1, 2, and peptide transporter 1 are highly expressed on the basolateral side in the small intestine, whereas OCT1-3 expression occurs primarily on the apical side.

Gastrointestinal tract absorption of anticancer drugs and medicines is typically measured using liquid chromatography/mass spectrometry (LC/MS) [11]. Although LC/MS requires liquid samples, such as blood or urine to measure gastrointestinally absorbed anticancer drugs and medicines, such liquid samples are affected by not only gastrointestinal tract absorption but also distribution throughout the body. Therefore, the purpose of this study was to estimate gastrointestinal tract absorption of cationic anticancer drugs and medicines via whole-body imaging following oral [^123^I]MIBG administration. After gastrointestinal tract absorption, cationic anticancer drugs and medicines primarily accumulate in the liver for metabolism and/or excretion via the urine and/or feces. It may be possible to estimate gastrointestinal tract absorption by focusing on drug-associated radioactivity in the liver and/or bladder.

## 2. Materials and Methods

### 2.1. Materials

[^123^I]MIBG (>95% of the radiochemical purity) was purchased from FUJIFILM Toyama Chemical Co., Ltd. (Tokyo, Japan). [^3^H]Methyl-4-phenylpyridinium (2.96 TBq/mmol) and *p*-[^14^C]aminohippuric acid (2.04 GBq/mmol) were purchased from American Radiolabeled Chemicals Inc (St. Louis, MO, USA) and PerkinElmer Inc. (Waltham, MA, USA). For in vivo study, cimetidine was purchased from Nacalai-Tesque (Kyoto, Japan). Dextran sodium sulfate (DSS, MW 36,000–50,000 Da) was purchased from Tokyo Chemical Industry Co., Ltd. (Tokyo, Japan). OCTN1/2 antibody (H-9) was purchased from Santa Cruz (Dallas, TX, USA).

### 2.2. Human Embryonic Kidney (HEK)293 Cells for SLC Transporters

SLC transporters were investigated using HEK293 cells expressing OATP2B1 (*SLCO2B1*, NM_007256), OCTN1 (*SLC22A4*, NM_003059), OCTN2 (*SLC22A5*, NM_003060), OCT1 (*SLC22A1*, NM_003057), OCT2 (*SLC22A2*, NM_003058), and OCT3 (SLC22A3, NM_021977). Briefly, HEK293 cells were transfected with plasmids encoding the respective transporters and then selected using appropriate antibiotics to establish HEK293/OATP2B1, HEK293/OCTN1, HEK293/OCTN2, HEK293/OCT1, HEK293/OCT2, HEK293/OCT3, and mock control cell lines. All cell lines were cultured at 37 °C and 5% CO_2_ in Dulbecco’s modified Eagle’s medium (Wako Pure Chemical Industries Ltd., Osaka, Japan) including 10% (*v*/*v*) fetal bovine serum (Life Technologies, Carlsbad, CA, USA), 100 μg/mL streptomycin, and 100 U/mL penicillin.

### 2.3. Analysis of [^123^I]MIBG Uptake by HEK293 Cells

Expression of OATP2B1 was confirmed in HEK293 cells using *p*-[^14^C]aminohippuric acid, and expression of OCTN and OCT was confirmed using [^3^H]methyl-4-phenylpyridinium [3,12]. One day before the uptake experiments, HEK293 cells expressing the respective SLC transporter were seeded at 4 × 10^5^ cells/well in 12-well plastic plates. Cells of each type were pre-incubated for 10 min in modified Hank’s balanced salt solution (MHBS) and then incubated with [^123^I]MIBG (37 kBq) for 5 min (*n* = 4), after which the cells were detached from the tissue culture plate using 0.25% trypsin-EDTA solution (Sigma-Aldrich, St. Luis, MO, USA). The radioactivity associated with the cells was measured using a gamma counter. Cellular protein content was measured using a bicinchoninic acid protein assay kit (Thermo Fisher Scientific Inc., Waltham, MA, USA) with bovine serum albumin as the standard. Uptake of [^123^I]MIBG by HEK293/OATP2B1, HEK293/OCTN1, HEK293/OCTN2, HEK293/OCT1, HEK293/OCT2, and HEK293/OCT3 cells was compared with uptake by mock control HEK293 cells. The effects of the OCTN and OCT inhibitor cimetidine [13,14] and OCTN inhibitor L-carnitine [15] were assessed by incubating HEK293 cells in a medium containing [^123^I]MIBG with and without 1 mM cimetidine and L-carnitine in MHBS. Uptake of [^123^I]MIBG by HEK293/OCTN1, HEK293/OCTN2, HEK293/OCT1, HEK293/OCT2, and HEK293/OCT3 cells was examined using this assay method, with [^123^I]MIBG uptake shown as % injected dose (%ID)/g protein.

### 2.4. DSS-Induced Experimental Colitis Mice

Animal experiments were approved by the Kanazawa University Committee for the Care and Use of Laboratory Animals (AP-194046) and were carried out in accordance with the guidelines for the Care and Use of Laboratory Animals. Male mice (4 weeks old, 18.9 ± 1.3 g, SLC Inc., Hamamatsu, Japan) were permitted free access to drinking water containing 2% (*w*/*v*) DSS from days 1 to 8 and days 22 to 29 and free access to DSS-free water from days 8 to 22 and days 29 to 31 [16]. Change in body weight was assessed every two days. Hematoxylin and eosin (HE) staining along with anti-OCTN1/2 (H9) immunohistochemical analysis were performed on specimens of tissue from the small and large intestines, liver, and kidney.

### 2.5. Biological Distribution of [^123^I]MIBG in DSS-Induced Experimental Colitis Mice

A total of 12 normal ddY mice (male, 9 weeks old, 33.7 ± 2.0 g) and 12 DSS-induced experimental colitis mice (male, 9 weeks old, 31.9 ± 2.4 g) were fasted overnight before experiments, with water supplied *ad libitum*. Each mouse with a clip on the penis to prevent urination was then intragastrically injected with approximately 185 kBq of [^123^I]MIBG via oral administration using feeding needles (Fuchigami). At 5, 10, 30, and 60 min after injection, the mice were euthanized under isoflurane (*n* = 3 per time point) after blood was sampled via cardiocentesis, and the following tissues were collected: thyroid, heart, stomach, liver, kidney, and bladder. The tissues were then weighed, and the radioactivity in the weighed tissue samples was measured using a gamma counter (AccuFLEX γARC-7010, Hitachi, Ibaraki, Japan). Data are expressed as %ID per g wet tissue (%ID/g tissue).

### 2.6. Single-Photon Emission Computed Tomography (SPECT) Imaging of [^123^I]MIBG in Normal and DSS-Induced Experimental Colitis Mice

Normal ddY mice (male, 9 weeks old, 37.4 ± 1.5 g, *n* = 5) and DSS-induced experimental colitis mice (male, 9 weeks old, 33.2 ± 2.9 g, *n* = 5) were intragastrically injected with [^123^I]MIBG (23.5 ± 2.0 MBq) under isoflurane gas anesthesia. SPECT acquisition was started 5 min after intragastric injection using a VECTor^+^ system (MILabs, Utrecht, The Netherlands). The acquisition data were reconstructed using the ordered subset expectation maximization method with 16 subsets and 6 iterations including resolution, attenuation and scatter correction. In the other reconstruction parameters, the voxel size was selected to 0.8 × 0.8 × 0.8 mm^3^ and a 1.0-mm Gaussian filter was applied as post-reconstruction smoothing filtering. For imaging analysis, the medical image data analysis software programs Pmod (ver. 3.7, PMOD Technologies LLC, Zurich, Switzerland) and Amide (exe. 1.0.4-1) were used [17]. SPECT images were obtained at 5–10 min, 30–35 min, and 60–65 min after oral administration of [^123^I]MIBG. Time-activity curves for the thyroid, heart, stomach, liver, kidney, and bladder were generated from the SPECT images, and coronal images are displayed as similar section images.

### 2.7. Biological Distribution of [^123^I]MIBG in the Presence/Absence of Cimetidine

One day before the experiments, a total of 24 ddY mice (male, 9 weeks old, 31.7 ± 1.8 g) were fasted with no food overnight, but water supplied *ad libitum*. The mice were then intragastrically injected with approximately 185 kBq of [^123^I]MIBG with or without cimetidine via oral administration using feeding needles (Fuchigami). Each mouse with a clip on the penis to prevent urination was then intragastrically injected with approximately 185 kBq of [^123^I]MIBG via oral administration using feeding needles (Fuchigami). At 5, 10, 30, and 60 min after injection, the mice were euthanized under isoflurane (*n* = 3 per time point) after blood was sampled via cardiocentesis, and the following tissues were collected: thyroid, heart, stomach, liver, kidney, and bladder. The tissues were then weighed, and the radioactivity in the weighed tissue samples was measured using a gamma counter (AccuFLEX γARC-7010, Hitachi, Ibaraki, Japan). Data are expressed as %ID per g wet tissue (%ID/g tissue). For studies with cimetidine loading (an OCT inhibitor with a high safety level in humans), ddY mice were orally administrated with a mixture of [^123^I]MIBG and cimetidine (approximately 7.5 μg/g mouse body weight in 100 μL of saline [18]), and the biological distribution analyses were performed using the same protocol used for control mice.

### 2.8. Statistical Analysis

*P*-values were analyzed using the two-tailed paired Student’s *t*-test for comparisons between two groups and analysis of variance and the Tukey test for comparisons among three groups using GraphPad Prism 8 statistical software (GraphPad Software, Inc., La Jolla, CA, USA) after normality testing using the Kolmogorov-Smirnov test. A *p* < 0.01 or 0.05 was considered as a significant difference.

## 3. Results

**Figure 1:**
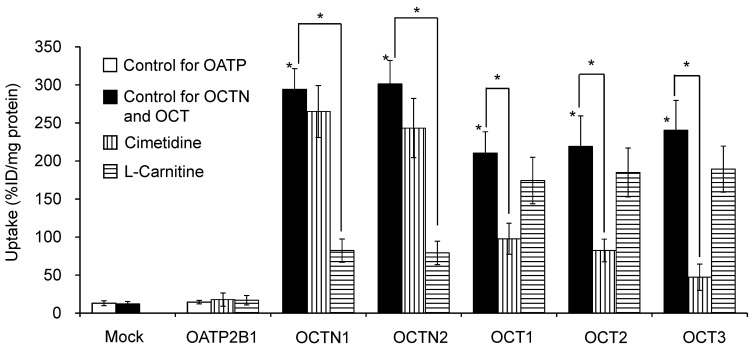
Uptake of [^123^I]MIBG by human embryonic kidney (HEK)293 cells expressing a solute carrier transporter (*n* = 4). Before the experiments with [^123^I]MIBG, the function of SLC transporters in HEK293 cells was confirmed using ^3^H- or ^14^C-substrates. Uptake of the ^3^H-labeled substrates into HEK293 mock cells as control cells is 107.1 ± 18.5%ID/g protein for HEK-Mock cells for organic anion transporting polypeptide (OATP), 84.9 ± 22.7%ID/g protein for HEK-Mock cells for organic cation/carnitine transporter (OCTN) and organic cation transporter (OCT). On the other hand, uptake of the ^3^H- or ^14^C-labeled substrates into HEK293 cells expressing a SLC transporter is as follows: 363 ± 38.6%ID/g protein for HEK293-OATP2B1, 387 ± 44.1%ID/g protein for HEK293-OCTN1, 379 ± 41.6%ID/g protein for HEK293-OCTN2, 403 ± 48.1%ID/g protein for HEK293-OCT1, 429 ± 58.5%ID/g protein for HEK293-OCT2, and 346 ± 37.1%ID/g protein for HEK293-OCT3. [^123^I]MIBG uptake is significantly higher in HEK293/OCTN1, HEK293/OCTN2, HEK293/OCT1, HEK293/OCT2, and HEK293/OCT3 cells than in mock cells. Cimetidine inhibits HEK293/OCT1, HEK293/OCT2, and HEK293/OCT3, and L-carnitine inhibits HEK293/OCTN1 and HEK293/OCTN2. * *p* < 0.01 vs. mock cells and HEK293 cells loaded with both inhibitors.

Figure 1 shows the uptake of [^123^I]MIBG by HEK293 cells expressing different SLC transporters and the inhibition of uptake by cimetidine and L-carnitine. The function of the SLC transporters in HEK293 cells was confirmed using *p*-[^14^C]aminohippuric for OATP and [^3^H]methyl-4-phenylpyridinium for OCTN and OCT. [^123^I]MIBG uptake was significantly higher in HEK293/OCTN1, HEK293/OCTN2, HEK293/OCT1, HEK293/OCT2, and HEK293/OCT3 cells than in mock cells. Uptake of [^131^I]MIBG by HEK293/OCT1, HEK293/OCT2, and HEK293/OCT3 cells was significantly inhibited only by cimetidine, whereas L-carnitine significantly inhibited [^131^I]MIBG uptake by HEK293/OCTN1 and HEK293/OCTN2 cells.

The biodistribution in normal mice, DSS-induced experimental colitis mice, and mice with oral loading of cimetidine with [^123^I]MIBG is shown in Table 1. In all mice, [^123^I]MIBG accumulated maximally in the stomach and kidney at 5 min and was then washed out. The accumulation of [^123^I]MIBG in the liver and bladder increased gradually over time. There was no diiodine because of little thyroid accumulation. Compared with normal mice, both DSS-induced experimental colitis mice and mice with cimetidine loading showed significantly lower accumulation in the blood at 5, 10, and 30 min, in the heart at 5, 10, and 30 min for DSS-induced experimental colitis mice and 5 min for mice with cimetidine loading, in the liver at all time points for DSS-induced experimental colitis mice and at 5 and 10 min for mice with cimetidine loading, and in the bladder at all time points for DSS-induced experimental colitis mice and at 5 and 10 min for mice with cimetidine loading.

**Figure 2:**
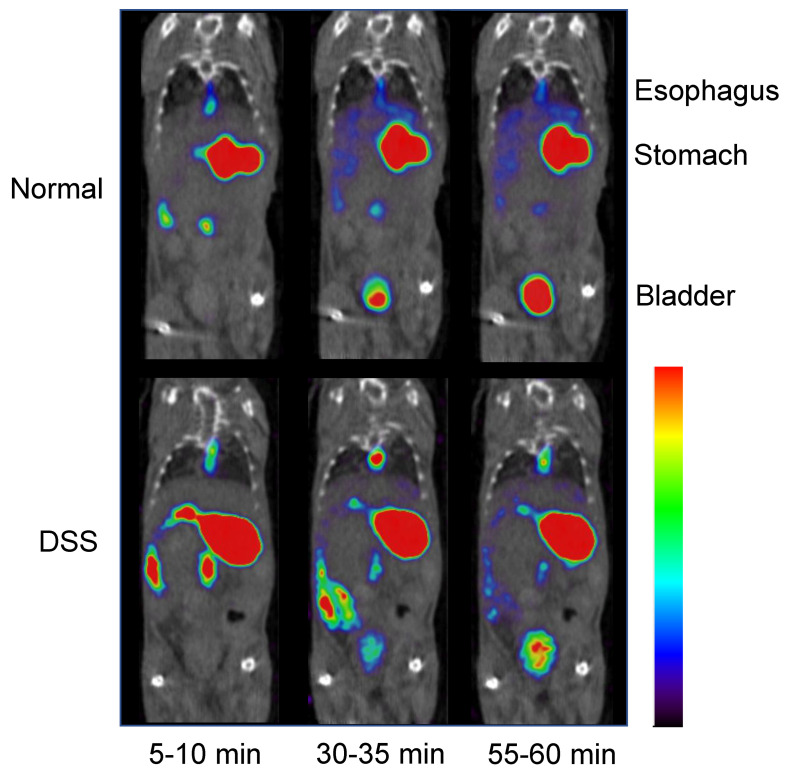
Representative [^123^I]MIBG single-photon emission computed tomography (SPECT) imaging in normal and dextran sodium sulfate (DSS)−induced experimental colitis mice at 5–10 min, 30–35 min, and 60–65 min after oral administration of approximately 20 MBq of [^123^I]MIBG. DSS-induced experimental colitis mice show decreased accumulation of [^123^I]MIBG in the bladder at 30–35 min and 60–65 min after oral [^123^I]MIBG administration and increased accumulation in the small intestine at 5–10 min and 30–35 min after oral [^123^I]MIBG administration.

SPECT images of [^123^I]MIBG in normal and DSS-induced experimental colitis mice at 10, 30, and 60 min after oral administration of [^123^I]MIBG are shown in Figure 2. A visually notable difference was observed in the bladder between normal and DSS-induced experimental colitis mice. Accumulation of [^123^I]MIBG increased in the small intestine at 5–10 min and 30–35 min after oral administration.

**Figure 3:**
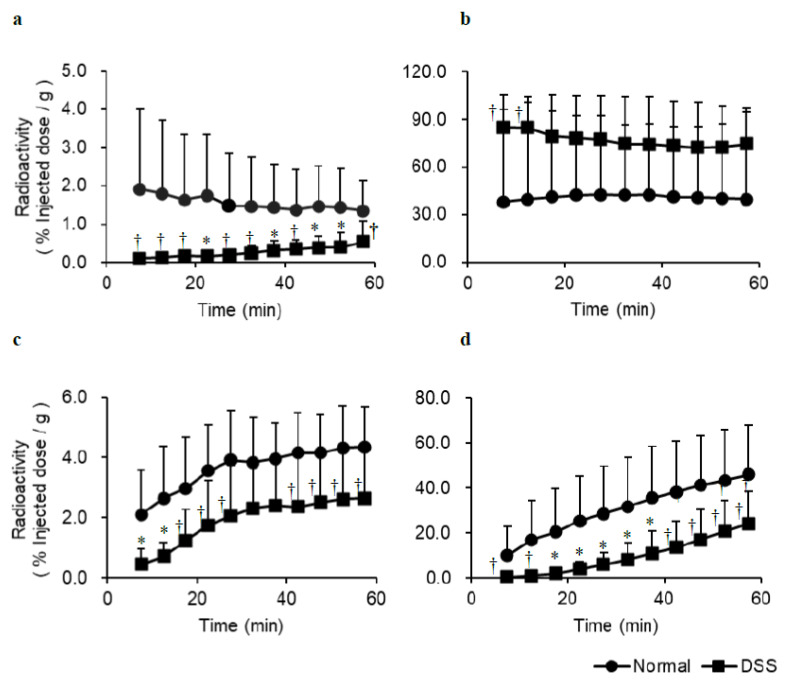
Time activity curves for the heart (**a**), stomach (**b**), liver (**c**), and bladder (**d**) in SPECT images of control and DSS-induced experimental colitis mice orally injected with [^123^I]MIBG. In DSS-induced experimental colitis mice, accumulation of [^123^I]MIBG is significantly lower in the heart, liver, and bladder than in normal mice, whereas accumulation is higher in the stomach in the early time points after oral [^123^I]MIBG administration. * *p* < 0.01 and ^†^ *p* < 0.05 between normal and DSS model mice.

Time-activity curves generated from the SPECT images of control and DSS-induced experimental colitis mice orally injected with [^123^I]MIBG is shown in Figure 3. In DSS-induced experimental colitis mice, accumulation of [^123^I]MIBG was significantly lower in the heart, liver, and bladder compared with normal mice, whereas [^123^I]MIBG accumulation was higher in the stomach at the early time points after oral administration.

**Figure 4:**
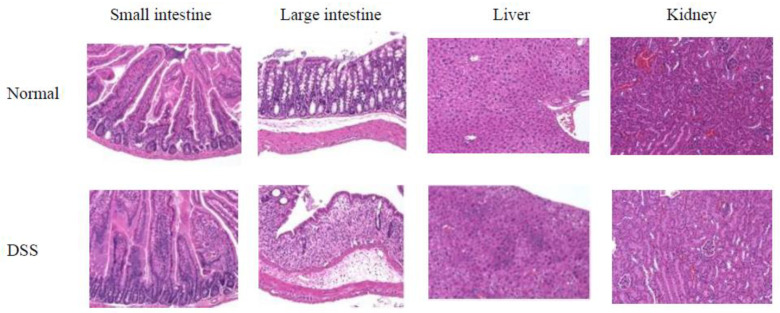
Anti-OCTN1/2 immunohistochemical analysis in the small intestine, large intestine, liver, and kidney. Purple indicates expression of OCTN1/2. The DSS-induced experimental colitis mice show relatively lower expression of OCTN1/2 than control mice in the small and large intestine. DSS has no effect on the liver and kidney.

HE staining in conjunction with anti-OCTN1/2 immunohistochemical analysis was performed on tissue from the small intestine, large intestine, liver, and kidney (Figure 4). The DSS-induced experimental colitis mice exhibited lower OCTN1/2 expression (purple dots) than control mice in the small and large intestines. DSS had no notable effects on the liver and kidney.

## 4. Discussion

In this study, [^123^I]MIBG was administered orally to estimate the gastrointestinal tract absorption of cationic anticancer drugs and medicines in the small intestine. This is important because individual patients can exhibit differences in gastrointestinal tract absorption, which can affect the activity of cationic anticancer drugs and medicines. An imaging method that enables estimation of gastrointestinal tract absorption of cationic anticancer drugs and medicines in the small intestine would be a very useful clinical tool.

[^123^I]MIBG is a water-soluble, cationic radiopharmaceutical that is taken up by various tissues via OCT [3,4]. We hypothesized that OCTN1/2 and OCT1-3 function primarily at the small intestinal epithelial cell membrane in the uptake of cationic drugs and medicines [9]. Indeed, [^123^I]MIBG exhibited a high affinity for OCTN1/2 and OCT1-3 (Figure 1). We, therefore, expect that oral administration of [^123^I]MIBG would enable the estimation of gastrointestinal tract absorption of cationic drugs and medicines in the small intestine. DSS-induced experimental colitis mice exhibit variations in the expression of OCTN, mainly OCTN1, at the basolateral side on the membrane of small intestinal epithelial cells [16]. Changes in the expression levels of OCTN1/2 induced by DSS were evaluated through anti-OCTN1/2 immunohistochemical analysis (Figure 4) because OCTN on the basolateral side is more important than OCT on the apical side in the small intestine to estimate gastrointestinal tract absorption following oral administration of [^123^I]MIBG. There was an increase in the number of purple dots indicating expression of OCTN1/2 in the small and large intestines of DSS-induced experimental colitis mice compared with normal mice. In addition, the collapse of the mucosal structure and infiltration of inflammatory cells was confirmed in both the small and large intestines. Thus, DSS-induced experimental colitis mice showed relatively increased expression of OCTN1 and OCTN2 compared to control mice.

Visual inspection of [^123^I]MIBG SPECT images (Figure 2) indicated reduced accumulation in the bladder of DSS-induced experimental colitis mice compared with normal mice. Time-activity curves of oral [^123^I]MIBG administration (Figure 3) indicated significantly lower accumulation of [^123^I]MIBG in the heart, liver, and bladder of DSS-induced experimental colitis mice compared with normal mice. In contrast, accumulation was higher in the stomach, because DSS reduces gastrointestinal tract absorption via OCTN1/2 at the basolateral side on the small intestinal epithelial cell membrane.

In the biodistribution study of oral [^123^I]MIBG administration in normal mice, DSS-induced experimental colitis mice, and cimetidine loading mice (Table 1), a significant decrease in radioactivity was observed in the blood, heart, liver, and bladder of DSS-induced experimental colitis mice and cimetidine loading mice compared with normal mice 5 min after oral [^123^I]MIBG administration, whereas the radioactivity increased in the stomach and kidney. Because the kidney expresses high levels of OCTN and OCT [5], the accumulation of radioactivity in the kidney should be increased in DSS-induced experimental colitis mice and mice with cimetidine loading. Thus, we considered that the cationic drug uptake activity of OCTN and OCT in the small intestine was inhibited by the effects of DSS and cimetidine, which inhibit OCT. As mentioned above, the accumulation of [^123^I]MIBG-associated radioactivity was also significantly lower in the heart, liver, and bladder in the DSS-induced experimental colitis mice compared with normal mice, as determined from time-activity curves generated based on [^123^I]MIBG SPECT images (Figure 2). A marked difference in uptake in the bladder between normal mice and DSS-induced experimental colitis mice was particularly notable.

Although intravenous administration of [^131^I]MIBG for radionuclide therapy has no serious hepatic or renal toxicities, side effects include mild nausea and transient elevation of arterial blood pressure [19]. Because [^123^I]MIBG has the same biological distribution as [^131^I]MIBG, oral administration of [^123^I]MIBG will not have serious side effects. As a limitation, the tight junction may have an effect on gastrointestinal tract absorption of orally administered [^123^I]MIBG [20]. However, OCTNs and/or OCTs were involved in gastrointestinal tract absorption of [^123^I]MIBG in a mouse study, because the effect of DSS to change to lower OCTN expression and that of cimetidine loading to inhibit OCT were confirmed (Table 1). Therefore, gastrointestinal tract absorption via OCTN and/or OCT in the small intestine can be estimated by measuring time-activity curves for the heart, liver, and bladder after oral [^123^I]MIBG administration.

## 5. Conclusions

[^123^I]MIBG imaging after oral administration can be used to estimate gastrointestinal tract absorption via OCTN and/or OCT in the small intestine based on analysis of time-activity curves for the heart, liver, and bladder.

## Figures and Tables

**Table 1:** Biodistribution of [^123^I]MIBG-associated radioactivity following oral administration in normal mice, DSS-induced experimental colitis mice, and mice with oral cimetidine loading.

Mice Types	Time after Oral [^123^I]MIBG Administration				
	Organ (%ID/g)	5 min	10 min	30 min	60 min
Normal mice	Blood	2.40 ± 0.41	2.11 ± 0.45	1.92 ± 0.52	1.61 ± 0.43
	Thyroid	0.05 ± 0.01	0.08 ± 0.03	0.08 ± 0.04	0.10 ± 0.04
	Heart	2.02 ± 0.36	1.97 ± 0.52	1.65 ± 0.48	1.31 ± 0.43
	Stomach	45.8 ± 3.22	41.9 ± 3.84	38.9 ± 3.11	36.1 ± 2.63
	Liver	1.74 ± 0.59	2.74 ± 0.62	3.21 ± 0.93	3.92 ± 0.81
	Kidney	1.14 ± 0.31	1.01 ± 0.38	0.72 ± 0.42	0.51 ± 0.39
	Bladder	10.1 ± 2.31	16.3 ± 3.05	21.2 ± 3.94	38.2 ± 4.31
DSS-induced experimental colitis mice	Blood	1.39 ± 0.19 *	1.73 ± 0.48 ^†^	1.58 ± 0.39 *	1.42 ± 0.43
	Thyroid	0.06 ± 0.03	0.09 ± 0.04	0.11 ± 0.03	0.10 ± 0.03
	Heart	0.91 ± 0.11 *	1.35 ± 0.22 *	1.21 ± 0.18 *	0.81 ± 0.11
	Stomach	85.1 ± 9.22 *	82.9 ± 10.84 *	78.5 ± 9.43 *	77.4 ± 9.89 *
	Liver	0.81 ± 0.15 *	1.25 ± 0.27 *	1.68 ± 0.31 *	2.12 ± 0.53 *
	Kidney	1.59 ± 0.47 ^†^	1.28 ± 0.41	0.88 ± 0.54	0.60 ± 0.41
	Bladder	2.41 ± 0.36 *	4.39 ± 0.85 *	13.8 ± 2.53 *	21.8 ± 3.89 *
Cimetidine loading mice	Blood	2.14 ± 0.30 ^†^	1.89 ± 0.23 ^†^	1.64 ± 0.34 ^†^	1.49 ± 0.41
	Thyroid	0.03 ± 0.01	0.05 ± 0.02	0.08 ± 0.03	0.10 ± 0.03
	Heart	1.75 ± 0.34 ^†^	1.91 ± 0.45	1.68 ± 0.50	1.40 ± 0.52
	Stomach	58.9 ± 4.44 ^†^	49.4 ± 4.93 ^†^	41.8 ± 4.12	38.8 ± 3.91
	Liver	1.39 ± 0.48 ^†^	2.46 ± 0.59 ^†^	3.18 ± 0.87	3.88 ± 0.97
	Kidney	1.51 ± 0.35 ^†^	1.10 ± 0.45	0.79 ± 0.46	0.58 ± 0.37
	Bladder	7.90 ± 1.53 ^†^	10.5 ± 2.95 ^†^	18.5 ± 4.51	32.9 ± 5.09

%ID/g indicates the percent injected dose per gram of tissue. Values are the mean ± standard deviation obtained from three mice. * *p* < 0.01 and ^†^ *p* < 0.05 compared with normal mice.
